# No Correlation Between Perception of Meaning and Positive Schizotypy in a Female College Sample

**DOI:** 10.3389/fpsyg.2020.01323

**Published:** 2020-06-12

**Authors:** Ubuka Tagami, Shu Imaizumi

**Affiliations:** ^1^Graduate School of Humanities and Sciences, Ochanomizu University, Tokyo, Japan; ^2^Institute for Education and Human Development, Ochanomizu University, Tokyo, Japan

**Keywords:** vision, meaning, signal detection, personality, schizotypy, schizophrenia, apophenia

## Abstract

We visually perceive meaning from stimuli in the external world. There are inter-individual variations in the perception of meaning. A candidate factor to explain this variation is positive schizotypy, which is a personality analogous to positive symptoms of schizophrenia (e.g., visual hallucination). The present study investigated the relationship between positive schizotypy, and the perception of meaning derived from meaningful and meaningless visual stimuli. Positive schizotypy in Japanese female undergraduates (*n* = 35) was assessed by the Cognitive-Perceptual dimension of the Schizotypal Personality Questionnaire. The participants were asked to report what they saw in noise-degraded images of meaningful objects (Experiment 1) and to respond whether the objects were meaningful (Experiment 2A) and which paired objects were meaningful (Experiment 2B). Positive schizotypy (i.e., Cognitive-Perceptual score) did not correlate with time to detect meaningful objects, and with false-alarm rates, sensitivity, and response criterion in the perception of meaning from meaningful and meaningless stimuli. These results were against our hypothesis and contradicted previous findings. The inconsistencies are discussed in terms of different methods (e.g., stimulus category) and conditions (e.g., paranormal beliefs).

## Introduction

In everyday life, we discriminate between useful and useless information by perceiving or extracting meaning from visual stimuli in the external world (Griffiths and Tenenbaum, 2007). The perception of meaning relies on our visual system as well as stochastic neural activity (Wild and Busey, 2004), stored representations (Gosselin and Schyns, 2003), and psychological states (Balcetis and Dunning, 2006). In addition to these intra-individual variations, there is also substantial inter-individual variation in the perception of meaning. Paranormal belief refers to a predisposition to believing paranormal phenomena which are physically impossible and difficult to explain through current science (Tobacyk and Milford, 1983). People with stronger paranormal beliefs are more likely to see something meaningful even in meaningless visual stimuli such as noise (Krummenacher et al., 2010; Riekki et al., 2013; Simmonds-Moore, 2014).

The perception of meaning may also be associated with positive symptoms in schizophrenia as characterized by, for example, visual and auditory hallucinations (Sass and Parnas, 2003) and paranormal beliefs (Shiah et al., 2014). Indeed, positive symptoms might originate from the abnormal perception of meaning because people in a pre-symptomatic stage of schizophrenia can feel that all events and stimuli present special meaning and suggestion (Brugger, 2001). However, detailed mechanisms of perception of meaning in patients with schizophrenia are yet to be fully understood, perhaps because it may be difficult to perform extensive experiments on patients due to reduced cognitive abilities, confounding effects of medication, and/or recruiting reasons.

Schizotypy, which is a psychological trait like positive and negative symptoms of schizophrenia, varies in healthy individuals and has been a candidate for analog study on perception in schizophrenia (Ettinger et al., 2015). In fact, some studies reported that positive schizotypy correlates with the perception of meaning as in positive symptoms of schizophrenia. People with higher positive schizotypy are more likely to perceive faces and objects in random visual noise (Partos et al., 2016), detect a meaningful word in non-words (Tsakanikos and Reed, 2005; Tsakanikos, 2006), and find meaningful relationships between unrelated events (Rominger et al., 2018). These studies suggest that positive schizotypy correlates with a higher frequency of false-positive responses in the perception of meaning. Findings from studies on schizotypy could also be beneficial for understanding the positive symptoms of schizophrenia.

To better understand the perception of meaning in schizotypy, we should examine whether positive schizotypy correlates not only with frequency of false-positive perception of meaning but also with sensitivity, latency, and confidence in the perception of meaning. It is assumed that positive schizotypy is associated with sensitivity in perception of meaning (e.g., Partos et al., 2016), and perhaps also with latency, given that higher schizotypy leads to slower reaction time in a visual attention task (Lenzenweger, 2001). However, confidence in perception of meaning could be associated with positive schizotypy independently of sensitivity, given that higher positive schizotypy can be associated with higher confidence but not with false-alarm rate in visual detection (Moritz et al., 2014) nor sensitivity in memory judgments (Corlett et al., 2009). These findings suggest that positive schizotypy may be associated with behavioral facets of the perception of meaning in a different (at times contradictory) way.

The present study aimed to conceptually replicate and extend previous findings showing a relationship between positive schizotypy and false-positive perceptions of meaning, by examining behavioral measures such as latency of detection of something meaningful in noise-masked images, confidence in the detection of a (false) meaning, and sensitivity in a two-choice task (i.e., meaningful-or-meaningless response). We employed two previous experimental paradigms. The first was a speeded detection task, where paranormal believers showed faster reaction time to detect a meaningful object in noise-masked scenic images than did non-believers (Simmonds-Moore, 2014). According to the previous study, in Experiment 1, participants immediately detected meaningful objects in noise-masked images and rated their confidence in their response. The second was a two-choice task where paranormal believers were more likely to judge meaningless (scrambled) faces as meaningful when they are presented in pairs of meaningful and meaningless stimuli (Krummenacher et al., 2010). It is possible that if two different stimuli are presented simultaneously, performance might be confounded by differences in stimulus properties (e.g., luminance distribution, shape) irrelevant to the difference in meaning. Therefore, Experiment 2A employed a modified version of this task, where participants judged whether a stimulus (i.e., a meaningful or meaningless object) was meaningful. Furthermore, for comparison, Experiment 2B employed another task using paired stimuli following Krummenacher et al. (2010). We hypothesized that people with higher positive schizotypy would detect a meaningful object faster in the noise-masked meaningful stimuli and rate higher confidence on their perception in Experiment 1. It was also hypothesized that they would find meaning in meaningless stimuli more frequently (i.e., higher false alarm rate), and show lower sensitivity in distinguishing meaningful and meaningless stimuli in Experiments 2A and B.

## Experiment 1

### Materials and Methods

#### Participants

*A priori* power analysis using G^∗^Power 3.1.9.4 (Faul et al., 2009) indicated that 34 participants were required on the assumption of a large effect size of 0.50 for correlation, statistical power of 0.90, and alpha of 0.05 (two-tailed). Forty Japanese female students voluntarily participated in this experiment. Five were excluded from the analysis; three did not complete the experiment, and two did not follow instructions. Data gathered from the remaining 35 students (mean age of 19.3 years, *SD* = 0.9, range = 18–21) were analyzed. All participants had normal or corrected-to-normal vision and no history of neurological or psychiatric illness. This study was approved by the ethics committee of the Graduate School of Humanities and Sciences, Ochanomizu University.

#### Measure of Schizotypy

A Japanese translation (Iijima et al., 2010) of the Schizotypal Personality Questionnaire (SPQ; Raine, 1991) was completed prior to the experiment. SPQ assesses schizotypal personality based on the criteria for schizotypal personality disorder defined in DSM-III-R. The present study employed the Cognitive-Perceptual composite score of the SPQ (see below) as an index of positive schizotypy, although a conceptual dissociation between schizotypal personality and schizotypy is still debated (Grant et al., 2018; Schultze-Lutter et al., 2019).

SPQ has 74-items and participants responded by using a yes/no scale. Yes and no responses are coded as scores of 1 and 0, respectively. Summed values served as subscale scores. We calculated 3 composite scores from 9 subscales of the SPQ according to Iijima et al. (2010): Cognitive-Perceptual, Interpersonal, and Disorganized. The Cognitive-Perceptual composite consisted of four subscales (e.g., ideas of reference, unusual perceptual experiences) that assess behaviors resembling positive symptoms in schizophrenia (Cronbach’s α = 0.873). We regarded the score of the Cognitive-Perceptual composite as an index of positive schizotypy. The Interpersonal composite consisted of three subscales (e.g., constricted affect) which assess behavior similar to negative symptoms (α = 0.771, note two items were excluded from calculation of α due to zero variance). The Disorganized composite consisted of two subscales (e.g., odd speech) which assess disorganized behavior (α = 0.846).

#### Stimuli and Apparatus

**FIGURE 1:**
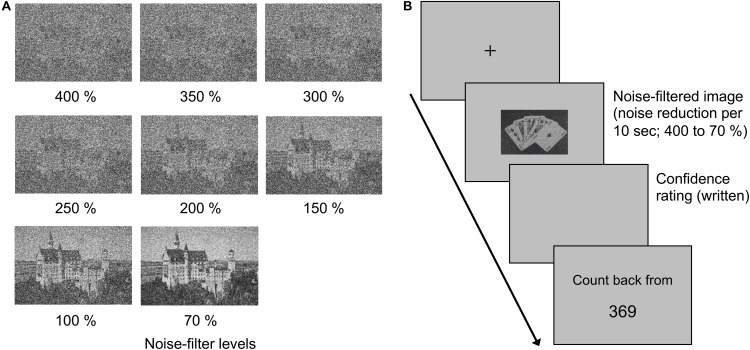
**(A)** A sample of noise-filtered images in Experiment 1. The value below each image denotes the degree of intensity of the added noise-filter. **(B)** Schematic of the task in Experiment 1.

According to Simmonds-Moore (2014), we chose 12 images of an object on background scene (e.g., statue, castle) from a royalty-free website^1^. Images were converted to grayscale. We cropped the images so that the main object was at the center of the image (400 × 600 pixels). Images were observed by participants from a distance of 57 cm and subtended approximately 10.2 × 15.2 degree of visual angle. To manipulate the visibility of the object, each image was degraded by applying 8 levels of noise filters (70, 100, 150, 200, 250, 300, 350, and 400%, Figure 1A) built into Adobe Photoshop CC 2019. Stimuli were presented on a 17-inch LCD monitor (FlexScan S170, EIZO). Participants responded using a QWERTY keyboard with their right hand. Stimulus presentation and response collection were controlled by PsychoPy 1.90.3 (Peirce et al., 2019) running on macOS 10.14.3.

#### Procedure

At the beginning of each trial (Figure 1B), a fixation cross was presented for 1 second, followed by a stimulus at the center of the screen. The noise-filter level was initially at 400% and reduced by one level every 10 s. Thus, stimuli were presented maximally for 80 s. Participants were asked to press the space key on a keyboard as soon as they saw something meaningful in the stimulus. The time interval between stimulus onset and keypress was recorded as the reaction time. After each keypress, the participants wrote down what they saw in the stimulus and rated their confidence on the accuracy of their response using a 7-point scale (1: “I have no confidence,” 4: “Neither,” 7: “I have confidence”). Null response on the object identification (e.g., “I do not know”) was allowed. All participants performed one trial with each stimulus in a randomized order. Between trials, participants performed a counting backward by threes for 20 s to prevent any carry-over from the previous trial. After the briefing, participants performed a practice trial and then 12 main trials. In the practice trial, a new stimulus, which was not used in main trials, was presented.

#### Data Analysis

If a stimulus presentation was over before a keypress response, we recorded the reaction time in that trial as 80 s (median of 1 trial, with interquartile range of 0–1.5). Trials where participants provided no answer or a “do-not-know” answer were excluded from the analysis. One trial was excluded from each of 5 participants. For each participant, reaction time and confidence rating scores were averaged.

To test the correlation between positive schizotypy and perception of meaning, we analyzed Pearson’s zero-order correlation coefficients between the SPQ’s Cognitive-Perceptual composite score, reaction time, and confidence rating. We also reported Kendall’s rank correlation coefficients when either or both variables were not normally distributed based on Shapiro-Wilk test. To quantify evidence of an alternative hypothesis (i.e., either positive or negative correlation) and a null hypothesis (no correlation), Bayesian correlation analyses were also performed with stretched beta prior of 1. For example, a Bayes factor in favor of a null hypothesis (BF_01_) of 3 indicates that the observation is three times more likely to occur under the null hypothesis than the alternative hypothesis. We interpreted BF_01_ larger than 3.00 as substantial evidence of null hypothesis, BF_01_ between 0.33 and 3.00 as insensitivity in distinguishing alternative and null hypotheses, and BF_01_ smaller than 0.33 as substantial evidence of alternative hypothesis (Dienes, 2014).

The three composite scores in SPQ were correlated with each other (*r*s > 0.393, *p*s < 0.020) due to the shared influence of neuroticism (Gross et al., 2014). Therefore, to remove influence of the Interpersonal and Disorganized composites and age on the relationship between positive schizotypy and behavioral measures, we performed multiple regression on each measure with the three SPQ composites and age as predictor variables. We forcibly entered the age in the first block and the Interpersonal and Disorganized composites in the second to check how much variance each predictor explained. The Cognitive-Perceptual composite was entered in the final block. Variance inflation factors were 1.94 for Cognitive-Perceptual, 1.26 for Interpersonal, 1.91 for Disorganized, and 1.11 for age, suggesting no substantial multicollinearity. Statistical analysis was performed using JASP 0.11.1 (JASP Team, 2019), except that multiple regression was performed using Jamovi 1.2.9 (Jamovi Project, 2020).

### Results and Discussion

**FIGURE 2:**
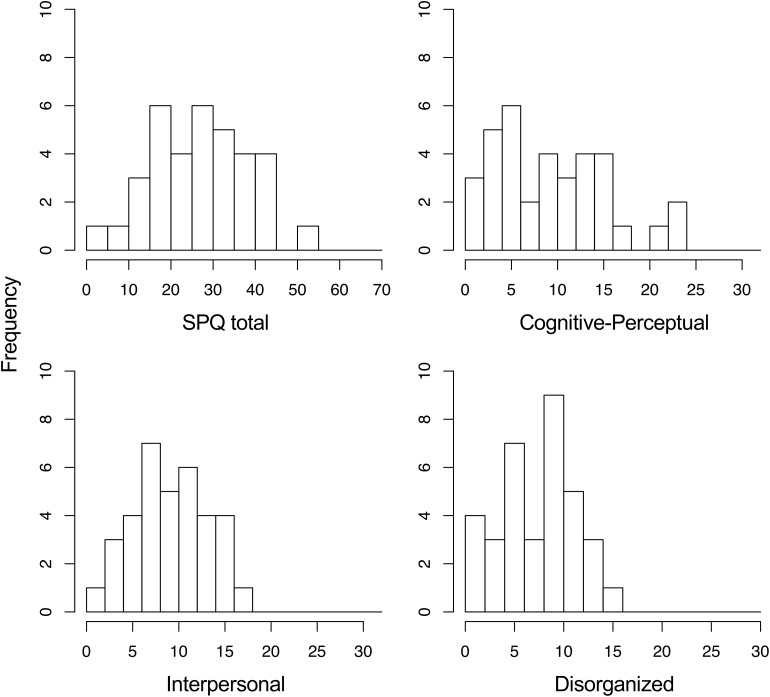
Distributions of the Schizotypal Personality Questionnaire (SPQ) scores (possible score ranges: total, 0–74; Cognitive-Perceptual, 0–33; Interpersonal, 0–25; Disorganized, 0–16).

Descriptive statistics are summarized in Table 1. The distributions of the SPQ scores are shown in Figure 2. The SPQ scores were compared with those found in a previous study using the same Japanese SPQ in a larger undergraduate sample (Iijima et al., 2010) (*n* = 558; 270 females, mean age of 19.4 years, *SD* = 1.1). The mean total SPQ scores were 25.1 (*SD* = 12.0) in Iijima et al. (2010) and 27.5 (*SD* = 11.4) in this study. Cohen’s *d* of 0.199 suggests that regarding overall schizotypal personality, the present sample did not substantially deviate from a representative Japanese student sample. Moreover, our Cognitive-Perceptual composite score (mean of 9.9) may also be comparable with the mean score (approximately 10.2) in Iijima et al. (2010), although its *SD* was not reported.

**TABLE 1:** Descriptive statistics in three experiments (*n* = 35).

	Measures	Mean	*SD*	Min	Median	Max	Shapiro-Wilk (*p*)
SPQ	Total	27.46	11.71	2	28	54	0.999
	Cognitive-Perceptual (positive schizotypy)	9.91	6.23	0	9	24	0.130
	Interpersonal	9.69	3.98	1	9	17	0.633
	Disorganized	7.86	3.96	0	9	15	0.343
Experiment 1	Reaction time (s)	45.20	9.96	26.45	47.45	66.70	0.367
	Confidence rating	4.13	0.87	1.50	4.08	5.92	0.356
Experiment 2A	Reaction time (ms)	656	117	505	633	1110	< 0.001
	False alarm rate	0.161	0.092	0.021	0.130	0.396	0.011
	*d*′	2.72	0.68	0.11	2.85	4.07	0.002
	c	–0.30	0.36	–0.73	–0.34	1.20	< 0.001
Experiment 2B	Reaction time (ms)	710	97	569	696	1054	0.001
	False alarm rate	0.391	0.199	0.083	0.417	0.875	0.200
	*d*′	1.93	0.71	0.38	1.94	3.15	0.504
	c	–0.65	0.33	–1.36	–0.59	–0.01	0.837

**SPQ, Schizotypal Personality Questionnaire.**

Positive schizotypy (i.e., Cognitive-Perceptual composite score) did not correlate with reaction time and confidence rating (|*r*|s < 0.103, n.s., BF_01_s > 4.03; Table 2). Multiple regression suggests that the Interpersonal and Disorganized composites and age did not affect reaction time and confidence rating (partial regression coefficients: *p*s > 0.231; coefficients of determination summarized in Table 3). Importantly, the Cognitive-Perceptual composite score did not show significant effects (*p*s > 0.327) even when it was entered in the model with these confounding predictors (for details, see Supplementary Table S1). These results were against our hypothesis positing that individuals with higher positive schizotypy are more likely to perceive a meaningful object and thus respond faster and be more confident in their responses. Although we followed a paradigm of Simmonds-Moore (2014) who showed exaggerated perception of meaning in paranormal believers, our findings were inconsistent with hers.

**TABLE 2:** Correlations between positive schizotypy (the Cognitive-Perceptual composite score) and behavioral measures in three experiments (Pearson’s *r* and Kendall’s tau; *n* = 35).

	Measures	Correlation with positive schizotypy		*P*	BF_01_
Experiment 1	Reaction time	*r*	–0.103	0.558	4.03
		tau	–0.089	0.459	3.48
	Confidence rating	*r*	0.054	0.758	4.54
		tau	0.122	0.317	2.73
Experiment 2A	**Reaction time**	*r*	–0.134	0.442	3.58
		tau	–0.131	0.279	2.54
	**False alarm rate**	*r*	–0.165	0.345	3.10
		tau	0.010	0.932	4.56
	***d*′**	*r*	0.304	0.076	1.05
		tau	0.109	0.369	3.04
	c	*r*	–0.096	0.584	4.12
		tau	–0.054	0.658	4.15
Experiment 2B	**Reaction time**	*r*	–0.069	0.695	4.42
		tau	0.052	0.669	4.18
	False alarm rate	*r*	–0.111	0.526	3.92
		tau	–0.079	0.520	3.70
	*d*′	*r*	0.136	0.434	3.55
		tau	0.096	0.425	3.32
	c	*r*	0.008	0.963	4.75
		tau	–0.010	0.932	4.56

**Some measures were non-normally distributed (bolded in the table). Parametric and non-parametric correlations were reported for descriptive purpose. BF_01_, Bayes factor in favor of null hypothesis.**

**TABLE 3:** Coefficients of determination (*R*^2^) for the three models of multiple regression on behavioral measures.

	Dependent variables	Predictors		
		Age	Age, Interpersonal, Disorganized	Age, Interpersonal, Disorganized, Cognitive-Perceptual
Experiment 1	Reaction time	0.036	0.043	0.046
	Confidence rating	0.005	0.037	0.068
Experiment 2A	Reaction time	<0.001	0.070	0.076
	False alarm rate	0.003	0.065	0.065
	*d*′	0.023	0.068	0.145
	c	0.024	0.093	0.135
Experiment 2B	Reaction time	0.008	0.027	0.034
	False alarm rate	<0.001	0.020	0.021
	*d*′	<0.001	0.015	0.021
	c	<0.001	0.019	0.019

**p-values for the coefficients ranged from 0.272 to 0.991 (see Supplementary Table S1). Predictors entered into the model forcibly.**

## Experiment 2A

### Materials and Methods

#### Participants

Thirty-five volunteers, whose data were analyzed in Experiment 1, participated in this experiment.

#### Stimuli and Apparatus

Stimuli were grayscale images displaying one object without a background. Each image subtended approximately 7.6 × 7.6 degrees (300 × 300 pixel). We generated 24 images for each of the meaningful, scrambled, and meaningless image categories. Meaningful images displayed a meaningful object (e.g., horse, frog, banana, tree, shoes, and car). Scrambled images depicted scrambled versions of the objects in the meaningful images. Meaningless images displayed objects without explicit meaning (e.g., ink blots, dirt on walls). The apparatus used was identical to Experiment 1.

#### Procedure

**FIGURE 3:**
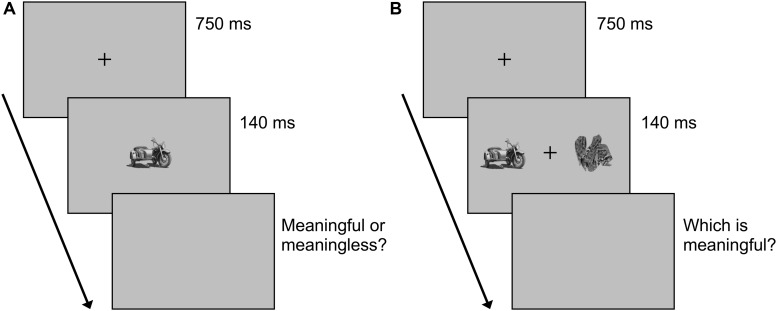
Schematic of the task in **(A)** Experiment 2A and **(B)** 2B.

This experiment was carried out on the same day as Experiment 1. At the beginning of the trial, a fixation cross was presented for 750 ms followed by an image for 140 ms (Figure 3A). Participants were asked to indicate whether the image displayed meaningful or meaningless objects by pressing the up or down arrow key on a keyboard. We informed participants that they would either see an object conveying a specific meaning or one with no specific meaning, but did not inform them of the scrambled objects. Participants performed one trial with 24 images in each of the three image categories (72 trials in total) in a randomized order. After the briefing, they performed 10 practice trials with images which had not been used in main trials and performed 72 main trials.

#### Data Analysis

Reaction time was averaged for each participant. A false alarm rate (i.e., “meaningful” response for scrambled and meaningless images) served as an index of exaggerated perception of meaning. Hit and false alarm rates were calculated collapsing three image categories were converted to an indices of sensitivity *d*′ and response criterion *c*, according to signal detection theory (Macmillan and Creelman, 2004). Reaction time, false alarm rate, *d*′ and *c* were tested for correlation with positive schizotypy (i.e., Cognitive-Perceptual composite score) and multiple regression as in Experiment 1.

### Results and Discussion

Since behavioral measures in this experiment were not normally distributed (Table 1), Kendall’s taus were reported. The Cognitive-Perceptual composite score did not correlate with reaction time, false alarm rate, *d*′ and *c* (|tau|s < 0.131, n.s.; Table 2). However, null correlation between Cognitive-Perceptual composite score and reaction time was inconclusive as suggested by BF_01_ of 2.54. The results of multiple regression suggest that Interpersonal, Disorganized, and age (partial regression coefficients: *p*s > 0.092), in addition to the Cognitive-Perceptual composite score (*p*s > 0.112), did not predict any of the behavioral measures (see also Table 3 and Supplementary Table S1).

Similar to Experiment 1, although inconclusive, there may be no relationship between positive schizotypy and time to detect meaning from visual stimuli. Importantly, our results indicate that positive schizotypy is not associated with false-positive responses, sensitivity, and response criterion in the perception of meaning in meaningful and meaningless stimuli. We further examined our hypothesis employing a choice task with paired stimuli in the next experiment.

## Experiment 2B

### Materials and Methods

Methods used were identical to those of Experiment 2A except for the following. We collected 48 new images for each of the meaningful, scrambled, and meaningless categories. In each trial, following the presentation of a fixation cross at the center of screen for 750 ms, a pair of two images was presented for 140 ms (Figure 3B). The center of each image was 7.5 degree left or right of the center of screen. Twenty-four pairs of meaningful and scrambled images, 24 pairs of meaningful and meaningless images, and 24 pairs of scrambled and meaningless images were presented in a randomized order. The presentation side of the images was balanced. After the presentation of paired images, participants were asked to judge the side where a meaningful image was presented by pressing the left or right arrow key. When participants found both meaningless, they pressed the up arrow key.

### Results and Discussion

Descriptive statistics and correlations are summarized in Tables 1, 2, respectively. As in Experiment 2A, positive schizotypy (i.e., Cognitive-Perceptual composite score) did not correlate with reaction time (tau = 0.052, n.s.), false alarm rate, *d*′, and *c* (|*r*|s < 0.136, n.s.), further supported by BF_01_s larger than 3.55. None of the predictors in the three regression models predicted any behavioral measures (partial regression coefficients: *p*s > 0.487; see also Table 3 and Supplementary Table S1). These results were against our hypothesis.

## General Discussion

### Summary of Results

The present study investigated the relationship between the perception of meaning in visual stimuli and positive schizotypy in a non-clinical Japanese sample. The Cognitive-Perceptual composite score in SPQ served as an index of positive schizotypy. We hypothesized that people with higher positive schizotypy perceive the meaning from visual stimuli faster and more frequently perceive meaning in meaningless stimuli (i.e., higher false alarm rate and lower sensitivity). However, the results of the three experiments were consistently contrary to our hypotheses based on both frequentist and Bayesian correlation analyses (except an inconclusive Bayesian correlation between positive schizotypy and reaction time in Experiment 2A). These null correlations between positive schizotypy and behavioral measures were replicated when the Interpersonal and Disorganized composites in SPQ and chronological age, in addition to positive schizotypy, were entered into regression models. Thus, positive schizotypy did not correlate with behavioral measures such as time to detect a meaningful object (Experiments 1 and 2B), confidence in the participants’ perceptions (Experiment 1), or sensitivity and response criteria to perceive meaning in meaningful and meaningless visual stimuli (Experiments 2A, B) based on signal detection theory. These null correlations suggest that positive schizotypy may not affect judgments of meaningful or meaningless stimulus and metacognitive confidence in them. However, it could highlight previously reported associations of positive schizotypy with the perception of meaning from meaningless stimuli (Tsakanikos and Reed, 2005; Partos et al., 2016) and illusory relationships between unrelated events (Rominger et al., 2018).

### Inconsistency With Previous Studies

The present findings were overall inconsistent with the previous studies. We propose three possible explanations for this discrepancy. The first is the difference in methods. In Experiment 1, positive schizotypy did not correlate with the perception of meaning, while in paranormal believers the relationship was found (Simmonds-Moore, 2014). We required our participants to provide one response about what they identified in each trial in order to focus more on the time needed to make the single reaction and the confidence with which it was done, while Simmonds-Moore (2014) did not limit the number of responses. To speculate, the number and/or variation of what one detects in meaningless stimulus, rather than time to initially detect, may reflect abnormal psychological traits. In Experiments 2A,B, positive schizotypy did not correlate with false-positive responses in meaning perception, while this was the case for paranormal beliefs (Krummenacher et al., 2010). We used more categories of objects (e.g., faces, animals, foods, and so on), while Krummenacher et al. (2010) used faces and words. It has been known that time to classify stimuli into given categories increases with category size (Pollack, 1963; Landauer and Freedman, 1968), suggesting that category size can interfere with cognitive information processing. To speculate, if this could be the case for perception of meaning, the larger category size in Experiment 2A,B may have affected meaningfulness judgment and consequently nullify the potential relationship between positive schizotypy and false-positive perception of meaning.

Second, we established hypotheses for all experiments from studies on paranormal believers (Krummenacher et al., 2010; Simmonds-Moore, 2014) because paranormal belief is one of the subscales of positive schizotypy assessed by SPQ and some reported that positive schizotypy also correlates with perception of meaning (Partos et al., 2016; Rominger et al., 2018). However, there may be a difference in the way perception of meaning is affected between paranormal beliefs and positive schizotypy. This notion is plausible because paranormal beliefs and positive schizotypy partially overlap and are explained by different surrounding constructs in the psychometric domain (Hergovich et al., 2008; Darwin et al., 2011). If the null correlations we found are the case, an exaggerated perception of meaning could be associated more with paranormal beliefs than with positive schizotypy.

Third, the schizotypy–a spectrum of personality that has features similar to schizophrenia–is not the same as clinical and pathological conditions. Thus, people with (relatively) high positive schizotypy may not necessarily exhibit hallucinatory experiences, including exaggerated perception of meaning (e.g., Sass and Parnas, 2003). It is possible that our sample might have fallen within a “mild” range where individuals do not show an abnormal perception of meaning, although the SPQ total and Cognitive-Perceptual composite scores in our sample did not substantially deviate from those of an earlier larger Japanese student sample (Iijima et al., 2010). While Krummenacher et al. (2010) and Simmonds-Moore (2014) employed a group-comparison approach (i.e., paranormal believers vs. non-believers), the present study employed a correlational approach. Given that a comparison between schizophrenic patients and healthy people can be a powerful method to detect the effects of psychiatric condition on abnormal perception (Panton et al., 2016), we expect group comparison to be suitable for our purpose.

### Limitations

Our participants were Japanese female university students. Thus, it will be difficult to generalize our findings to other ethnicities, genders, and age groups. Indeed, gender and age could be confounding factors. Although some studies showed no gender difference in schizotypy (Fossati et al., 2003), other studies reported that schizotypy score was higher in females than in males (Claridge and Hewitt, 1987). Females in particular, tend to have higher positive schizotypy than males, while males have higher negative schizotypy than females (Raine, 1992; Venables and Bailes, 1994). On the other hand, it has also been reported that adolescents show higher schizotypy than adults (Venables and Bailes, 1994). Further, there could be an interaction effect of gender and age on the structure of schizotypy (Ito et al., 2010). These potential effects of gender and age might influence the relationship between positive schizotypy and the perception of meaning. Further studies employing broader sample are needed.

Although our methods followed those of previous studies (Krummenacher et al., 2010; Simmonds-Moore, 2014), we did not fully control the physical characteristics of the stimuli (e.g., mean luminance and size). Future studies should employ more rigorously controlled methods (e.g., Partos et al., 2016) to better replicate and extend the present findings.

Since we investigated the perception of meaning only in vision, our findings cannot be generalized to other sensory modalities. In patients with schizophrenia, auditory hallucinations are more common than visual hallucinations (Mueser et al., 1990) and decreased perceptual sensitivity may be found in auditory and audiovisual, but not visual modalities (Mussgay and Hertwig, 1990). Given this, one might speculate that auditory perception of meaning is likely to reflect characteristics of heightened schizotypy and schizophrenia. Indeed, some studies have reported that positive schizotypy correlates with false-positive hearing such as voice perception from white noise (Barkus et al., 2007, 2011). Future studies should investigate how positive schizotypy relates to perception of meaning in meaningful or meaningless auditory stimuli.

The present study employed SPQ, which is a self-report measure of schizotypal personality disorder, as an index of schizotypy. However, schizotypal personality might be conceptually dissociated from schizotypy (Grant et al., 2018). It has been argued that schizotypal personality is an observable phenomenological entity that derives from schizotypy – a latent construct (Lenzenweger, 2015). Therefore, the Cognitive-Perceptual score of the SPQ might not adequately measure positive schizotypy and thus might have had less power to detect the relationship between positive schizotypy and perception of meaning in the present study. Thus far, we have only discussed trait-like schizotypy. However, as it has been additionally argued that variations in state measures of positive schizotypy (e.g., repeated measurement during experiments) could better explain abnormal false-positive perception than trait measures (Grant et al., 2014) such as SPQ, our experimental paradigm should be re-examined by assessing state and trait positive schizotypy.

## Conclusion

The present study of Japanese female undergraduates found no relationship between positive schizotypy and perception of meaning, especially in the visual detection of meaning in noise-masked meaningful stimuli, confidence in the detection, and discrimination between meaningful and meaningless visual stimuli. The null correlations simultaneously highlight the known effect of positive schizotypy on the detection of meaningful objects from meaningless stimuli (e.g., Partos et al., 2016) and the illusory perception of associations between events (Rominger et al., 2018). The present study can contribute to an accumulation of data for the elucidation of perceptions and symptoms in schizophrenia and schizophrenic spectrum.

## Data Availability Statement

All datasets generated for this study are included in the article/Supplementary Material.

## Ethics Statement

The studies involving human participants were reviewed and approved by the ethics committee of the Graduate School of Humanities and Sciences, Ochanomizu University. The patients/participants provided their written informed consent to participate in this study.

## Author Contributions

UT conceived the study and performed the experiments. UT and SI analyzed the data, wrote the manuscript, and approved the final version of the manuscript.

## Conflict of Interest

The authors declare that the research was conducted in the absence of any commercial or financial relationships that could be construed as a potential conflict of interest.

## Footnotes

1 https://pixabay.com
